# Rat model of attention-deficit hyperactivity disorder exhibits delayed recovery from acute incisional pain due to impaired descending noradrenergic inhibition

**DOI:** 10.1038/s41598-023-32512-9

**Published:** 2023-04-04

**Authors:** Takashi Suto, Daiki Kato, Ikuya Koibuchi, Yuki Arai, Jo Ohta, Tadanao Hiroki, Hideaki Obata, Shigeru Saito

**Affiliations:** 1 Department of Anesthesiology, Gunma University Graduate School of Medicine, 3-39-22 Showa-machi, Maebashi, Gunma 371-8511 Japan; 2 Department of Anesthesiology, Saitama Medical Center, Saitama Medical University, 1981 Kamoda, Kawagoe, Saitama 350-8550 Japan

**Keywords:** Psychiatric disorders, Neuroscience, Diseases

## Abstract

Chronic pain and attention-deficit hyperactivity disorder (ADHD) frequently coexist. However, the common pathology is still unclear. Attenuated noradrenergic endogenous analgesia can produce acute pain chronification, and dysfunction of noradrenergic systems in the nervous system is relevant to ADHD symptoms. Noxious stimuli-induced analgesia (NSIA) is measured to estimate noradrenergic endogenous analgesia in spontaneously hypertensive rats (SHR) as an ADHD model and control. Recovery of pain-related behaviors after paw incision was assessed. Contributions of noradrenergic systems were examined by in vivo microdialysis and immunohistochemistry. The SHR showed attenuated NSIA and needed a more extended period for recovery from acute pain. These results suggest ADHD patients exhibit acute pain chronification due to pre-existing attenuated noradrenergic endogenous analgesia. Immunohistochemistry suggests abnormal noradrenaline turnover and downregulation of the target receptor (alpha2a adrenoceptor). Standard ADHD treatment with atomoxetine restored NSIA and shortened the duration of hypersensitivity after the surgery in the SHR. NSIA protocol activated the locus coeruleus, the origin of spinal noradrenaline, of both strains, but only the control exhibited an increase in spinal noradrenaline. This result suggests dysfunction in the noradrenaline-releasing process and can be recognized as a novel mechanism of attenuation of noradrenergic endogenous analgesia.

## Introduction

Attention-deficit hyperactivity disorder (ADHD) is related to chronic pain in children and adults. The global prevalence of adults with ADHD is reported to be 2.5%^1^, but a higher frequency of coexisting ADHD is reported among patients with various types of chronic pain^2^. e.g., fibromyalgia (55/125, 44.7%)^3^, low back pain (19/60, 31.7%)^4^, and chronic pain in children (29/146, 19.9%)^5^. The pathology of ADHD is considered to involve altered noradrenergic modulation of brain function. Interestingly, drug treatments for ADHD and chronic pain are similar; noradrenaline (NA) reuptake inhibitors and alpha2-adrenergic agonists are used for both.

Problems in attention, hyperactivity, and impulsiveness are observed in ADHD patients. Spontaneously hypertensive rats (SHR), ordinally used as a rat model of hypertension, exhibit excessive locomotion in an open field test or elevated plus maze^6^, which suggests hyperactivity, and fewer spontaneous alterations in the Y-maze^7^, which reflects a lack of attention. SHR show impairment in short term memory^8^. Altered NA function in the central nervous system is a possible mechanism underlying these behaviors in SHR^9^. Standard treatments for human ADHD patients, atomoxetine^10^ or guanfacine^11^, reduce the ADHD-like behaviors of SHR. Therefore, SHR are considered as an animal model of ADHD.

The locus coeruleus (LC) is a central nucleus containing noradrenergic neurons that projects to various brain regions, including the prefrontal cortex and spinal cord^12^. Brain NA is involved in modulating working memory, attention, and motivation^13–15^, brain functions that are impaired in ADHD patients. In animal models of neuropathic pain, similar impairments in the noradrenergic modulation of brain function are observed, especially at late stages after nerve injury^16,17^. A common pathology might exist between the brain function of ADHD and that of chronic pain animals. ADHD and chronic pain may also share pathology in pain modulation. The LC of chronic neuropathic animals does not respond to painful stimulation, resulting in attenuation of descending noradrenergic pain inhibition^16^. However, alterations of LC activity in SHR have not been investigated. This study hypothesized that noradrenergic pain modulation would be altered by spinal or/and brain mechanisms in SHR. It may be crucial as a risk factor for acute pain chronification. Alternatively, it may be a proposal for a new mechanism of chronic pain.

To investigate endogenous pain modulation, including descending noradrenergic inhibition, we examined noxious stimuli-induced analgesia (NSIA) as a known type of conditioned pain modulation in SHR and compared it with control animals, Wister Kyoto (WKY).

We also repeatedly examined the pain threshold before and after the hind paw incision to examine the recovery period from acute pain in both strains. Intrathecal injection of an alpha2-adrenoceptor agonist or antagonist was used to examine whether SHR exhibit a different response to noradrenergic analgesics in acute pain conditions. To elucidate spinal and LC mechanisms, microdialysis was used to measure changes in the NA concentration in the spinal dorsal horn (ScDH) and medial prefrontal cortex after noxious stimulation induced by subcutaneous capsaicin injection. Phosphorylated extracellular signal-related kinase (pERK) and c-fos immunostaining in noradrenergic neurons in the LC were evaluated to observe differences in neuronal activation. Furthermore, to clarify the effects of ADHD treatment on pain modulation and pain threshold after paw incision, the SHR treated with the NA reuptake inhibitor, atomoxetine, were included in these experiments.

## Results

### Validation of ADHD-like behaviors of SHR

To validate SHR/Izm as an animal model of ADHD, open-field test was performed to evaluate the locomotor activity and anxiety. SHR-ATX group was included to examine the effects of standard ADHD treatment (atomoxetine, 0.3 mg/kg/day, 14 days). SHR and SHR-ATX showed longer locomotion during the test than control (1450 ± 584.7 vs. 1491 ± 221.5 vs. 1016 ± 311.0 px., Supplement Fig. 1A). One-way ANOVA revealed the significant effect (F 2,39 = 5.97, *P* = 0.0055) and post-hoc test showed a significant difference between SHR and control (*P* = 0.02). Repeated atomoxetine treatment did not alter the distance (SHR vs. SHR-ATX, *P* > 0.999). SHR and SHR-ATX showed longer duration spent in the center area than control (170 ± 198 vs. 743.4 ± 390 vs. 733.2 ± 357 frames, Supplement Fig. 1B). One-way ANOVA revealed the significant effect (F 2,39 = 14.15, *P* < 0.0001) and the post-hoc test showed a significant difference between SHR and control (*P* < 0.0001). Repeated atomoxetine treatment did not alter the duration in the center area (SHR vs. SHR-ATX, *P* > 0.999). SHR/Izm show hyperactive and impulsive behaviors that reflect ADHD-like symptoms. However, this dose of atomoxetine treatment did not alter the behaviors in our experiments, similar to the previous study^6^.

**Figure 1:**
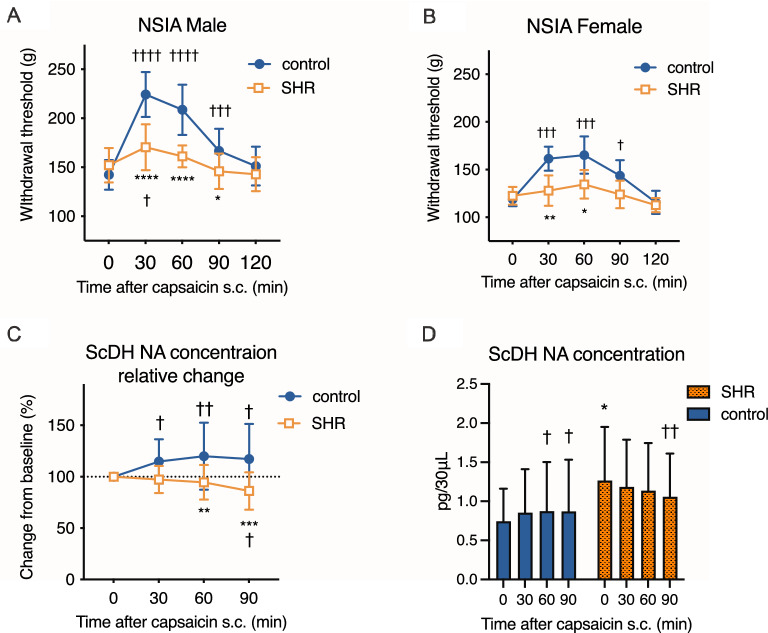
Noxious stimuli-induced analgesia (NSIA) in SHR is attenuated. Left forepaw injection of capsaicin raised the withdrawal threshold of the right hind paw of control from 30 to 90 min after injection. SHR, however, respond to capsaicin only at 30 min in male (**A**). Female SHR does not respond to capsaicin (**B**). Relative change in the noradrenaline concentration in the spinal dorsal horn after capsaicin injection was measured by microdialysis (**C**). Left forepaw injection of capsaicin increased the noradrenaline concentration in the spinal dorsal horn at 30, 60, and 90 min after injection. In SHR, however, the spinal dorsal horn NA concentration decreased from 60 and 90 min. The baseline spinal noradrenaline concentration from the microdialysate is higher in SHR (**D**). Mean ± SD, ††††; *p* < 0.0001 versus time 0, †††; *p* < 0.001 versus time 0, ††; *p* < 0.01 versus time 0, †; *p* < 0.05 versus time 0, ****; *p* < 0.0001 versus control, ***; *p* < 0.001 versus control, **; *p* < 0.01 versus control, *; *p* < 0.05 versus control, n = 16 male, 8 females for each strain (WKY-male:16, WKY-female:8, SHR-male:16, SHR-female:8), n = 20, 17 (C, D, male only). SHR: spontaneously hypertensive rats (ADHD model), control: Wister Kyoto.

Y-maze test was also performed to evaluate the locomotor activity and inattention or short term working memory. There were no differences between SHR or SHR-ATX and control in spontaneous alternation or alternate arm return (61.34 ± 7.1 vs. 69.78 ± 10.5 vs. 62.91 ± 12.4%., Supplement Fig. 2A, 28.13 ± 7.5 vs. 26.4 ± 10.2 vs. 30.56 ± 8.5%., Supplement Fig. 2B). One-way ANOVA revealed no significant effect (F 2,41 = 2.91, *P* = 0.065, F 2,41 = 0.82, *P* = 0.44). Repeated atomoxetine treatment did not alter these alternation behaviors. However, SHR showed more same arm return than control and SHR-ATX (1[0–1] versus 0 [1, 2] versus 0 [0–1] times, Supplement Fig. 2C). Kruskal–Wallis test revealed a significant effect (*P* = 0.0011), and multiple comparisons revealed SHR showed more same arm return than the control (*P* = 0.0039) and atomoxetine treatment reduced the time (SHR vs. SHR-ATX, *P* = 0.0038).

SHR and SHR-ATX showed more time of arm entry than control (SHR: 20 [19–24] versus SHR-ATX: 21.5 [19–23] versus control 16 [13–20]). Kruskal–Wallis test revealed a significant effect (*P* = 0.0024), and multiple comparisons revealed SHR showed more time of arm entry than control (*P* = 0.0059) and atomoxetine treatment did not reduce the time (SHR vs. SHR-ATX, *P* > 0.99). Our Y-maze results indicate that SHR/Izm show hyperactivity and inattention, and atomoxetine partially reduced the behaviors. The dose of atomoxetine which is effective on Y-maze scores in SHR is not reported, but the dose of our experiments is lower than the previously reported effective dose in mice^19^.

The open field and Y-maze test results suggest that SHR/Izm reflect ADHD-like symptoms the same as a previous study^7^, but they did not respond to atomoxetine. This may be because the dose of atomoxetine used in this study is less than previously reported effective doses on these tests.

### Noxious stimuli-induced analgesia (NSIA) was attenuated in SHR

To evaluate endogenous analgesia in SHR and control (WKY), we assessed NSIA. Capsaicin injection into the left forepaw was used as the painful stimulation, and the withdrawal threshold in the left hind paw was measured using the paw pressure test (Fig. 1A,B). A 2-way ANOVA revealed a significant main effect of strain (F 1,30 = 33.15, *p* < 0.0001) and interaction (F 4,120 = 18.22, *P* < 0.0001), and time after capsaicin (F 4,120 = 54.96, *P* < 0.0001). In females, a 2-way ANOVA revealed a significant main effect of strain (F 1,14 = 24.10, *p* = 0.00002), time after capsaicin (F 4, 56 = 21.72, *P* < 0.0001), and interaction (F 4, 56 = 6.388, *P* = 0.0003).

These results suggested that the withdrawal threshold was increased in control of both sexes from 30 to 90 min after capsaicin injection. However, the withdrawal threshold of SHR was increased only at 30 min in males. The differences between before and 30 min after capsaicin injection were smaller in SHR (Male: 18.4 ± 30.1 vs. 82.1 ± 30.5 g, *P* < 0.0001, η^2^ = 0.054, Female: 5.38 ± 16.2 vs. 42.5 ± 12.9 g, *P* = 0.0002, η^2^ = 0.65), suggesting that endogenous analgesia including descending noradrenergic inhibition is attenuated.

### Spinal microdialysis

To examine the effect of noradrenaline (NA) on NSIA, the NA concentration in the spinal dorsal horn (ScDH) was measured by in vivo microdialysis before and after capsaicin injection in male rats (Fig. 1C). We used males because the time course of NSIA is similar in both sexes. A 2-way ANOVA revealed significant main effects of strain (F 1,35 = 11.49, *P* = 0.0017) and interaction (F 3,105 = 7.557, *P* = 0.00001), but not time after capsaicin (F 3,105 = 1.717, *P* = 0.168), suggesting that capsaicin injection increased spinal NA by 25% or more only in control. In SHR, however, the NA concentration decreased after capsaicin injection. The absolute value of the spinal NA concentration prior to and after capsaicin injection is shown in Fig. 1D.

### Alterations of spinal noradrenergic systems

**Figure 2:**
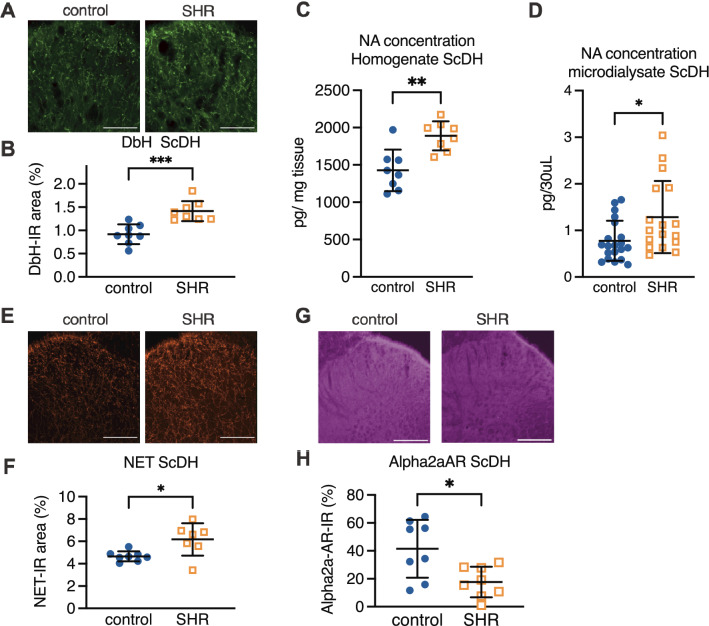
The noradrenergic systems in the spinal dorsal horn. Representative pictures of dopamine-β-hydroxylase (DbH) in the spinal dorsal horn from SHR and control (**A**). The percentage of DbH immunoreactive (IR) area is summarized, and SHR show a larger DbH-IR area than control (**B**). Noradrenaline concentration in the homogenate of the spinal is higher in SHR than in control (**C**). The concentration of spinal extracellular noradrenaline measured by spinal microdialysis prior to capsaicin injection is also higher in SHR than in control (**D**). Representative pictures of the noradrenaline transporter (NET) (**E**). SHR had a significantly greater NET-IR area than control (**F**). Representative pictures of the alpha2-adrenoceptor (Alpha2aAR) (**G**). SHR had a significantly lower Alpha2aAR-IR area (**H**). Mean ± SD, *; *p* < 0.05, **; *p* < 0.01, ***; *p* < 0.001, n = 8 each (**B**, **C**, **F**, **H**), n = 25, 19 (**D**), Scale bar = 200 µm. SHR: spontaneously hypertensive rats (ADHD model), control: Wister Kyoto, ScDH: spinal dorsal horn.

To reveal the alterations of noradrenergic pain inhibition of SHR more clearly, we investigated NA synthesis, reuptake, and target receptor in the spinal cord. Representative pictures of the NA synthesis enzyme, dopamine-β-hydroxylase (DbH), from the ScDH of SHR and control are shown in Fig. 2A. DbH-IR areas were greater in the SHR compared with control (1.41 ± 0.21 vs. 0.92 ± 0.21%, *P* = 0.0004, η^2^ = 0.61) (Fig. 2B), suggesting greater NA synthesis in SHR. The NA concentration in spinal cord homogenate was actually higher in SHR (1889 ± 194 vs. 1427 ± 289 pg/mg tissue, *P* = 0.0018, η^2^ = 0.51, Student’s t-test) (Fig. 2C). Furthermore, the basal extracellular NA concentration in SHR measured by in vivo spinal microdialysis was also higher than in control (1.29 ± 0.77 vs. 0.78 ± 0.43 pg/30μl, *P* = 0.016, η^2^ = 0.15) (Fig. 2D). Conversely, the noradrenaline transporter (NET) -IR areas were greater in the ScDH of SHR compared with control (6.17 ± 1.44 vs. 4.67 ± 0.44%, *P* = 0.014, η^2^ = 0.38) (Fig. 2E,F), suggesting greater NA reuptake.

The representative pictures of target receptors of NA, alpha2-adrenoceptors (Alpha2aAR), are shown in Fig. 2G. The Alpha2aAR-IR area was smaller in SHR than in control (17.72 ± 10.98 vs. 41.74 ± 20.74%, *P* = 0.012. η^2^ = 0.37) (Fig. 2H).

Noradrenergic systems in mPFC of SHR are shown in supplement Fig. 3. The same as the spinal cord, capsaicin injection to the forepaw did not increase the noradrenaline concentration in the mPFC of SHR (supplement Fig. 3F).

**Figure 3:**
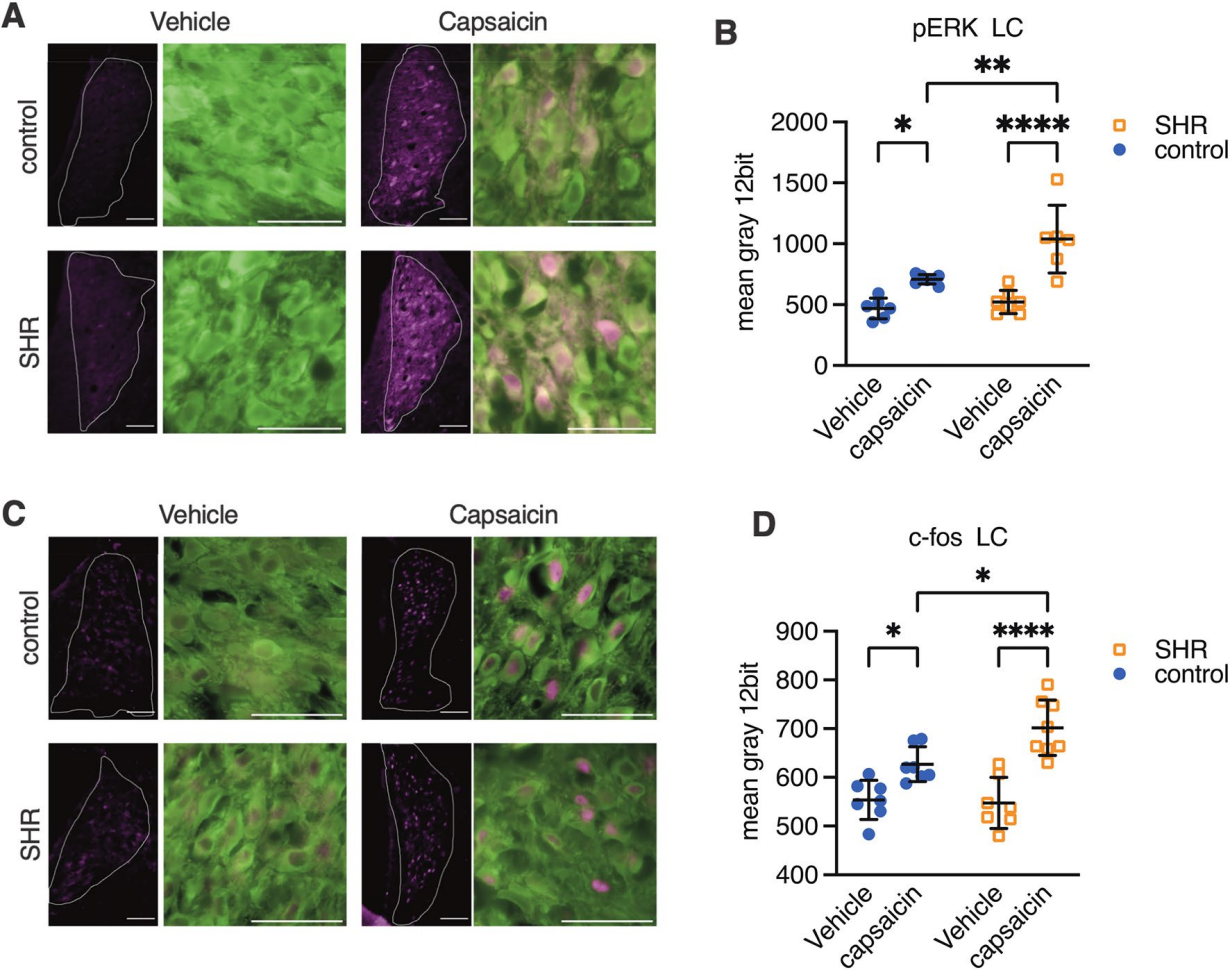
Capsaicin-evoked activation of locus coeruleus noradrenergic neurons. Representative pictures of pERK-immunoreactivity (IR) in the locus coeruleus (LC) area are shown in (**A**). 30 min after capsaicin or vehicle injection to the left forepaw, the brain tissue was collected and stained. Low-magnification images are shown on the left. The white dot lines show the LC area determined by dopamine beta hydroxylase (DbH) -IR. pERK-IR pixels are shown in magenta. In the right pictures, DbH-IR is presented in green and pERK-IR is presented in magenta. The mean gray value of pERK-IR from each noradrenergic neuron was measured, and the average value was calculated for each rat (**B**). There are no statistical differences in pERK between the strains after vehicle injection. Capsaicin injected animals show higher IR of pERK than vehicle injected animals. Representative pictures of c-fos-IR in the LC area 60 min after capsaicin or vehicle injection are shown in (**C**). There are no statistical differences in c-fos between the strains after vehicle injection. Capsaicin injected animals show higher IR of c-fos than vehicle injected animals. Mean ± SD, *; *p* < 0.005, **; *p* < 0.01, ****; *p* < 0.0001. n = 8 each. Dot line: LC is determined by DbH-IR, Scale bar = 200 µm (left), 100 µm (right). SHR: spontaneously hypertensive rats (a model of attention deficit hyperactive disorder: ADHD), control; Wister Kyoto.

### Capsaicin induced locus coeruleus activation

The spinal microdialysis results suggested that the locus coeruleus (LC) of SHR does not respond to noxious stimulation. Considering that the NA concentration and NET expression were higher in the ScDH of SHR than that of control, it was necessary to verify the activity of LC noradrenergic neurons, which project to the ScDH. Expression of pERK and c-fos in LC neurons prior to and after capsaicin stimulation were thus examined by immunohistochemistry (Fig. 3A).

Analysis of pERK expression by 2-way ANOVA revealed significant main effects of strain (F 1,22 = 10.70, *P* = 0.0035), capsaicin (F 1,22 = 41.74, *P* < 0.0001), and interaction (F 1,22 = 5.547, *P* = 0.0278) (Fig. 3A, B). Analysis of c-fos expression also revealed significant main effects of capsaicin (F 1,25 = 41.21, *P* < 0.0001) and interaction (F 1,25 = 5.219, *P* < 0.0278), but not strain (F 1,25 = 3.706, *P* = 0.066) (Fig. 3C, D). Capsaicin increased pERK and c-fos in the LC of both SHR and control. Expression of both pERK and c-fos after capsaicin injection was stronger in SHR compared with control.

Treatment with the NA reuptake inhibitor atomoxetine restored NSIA in SHR.

**Figure 4:**
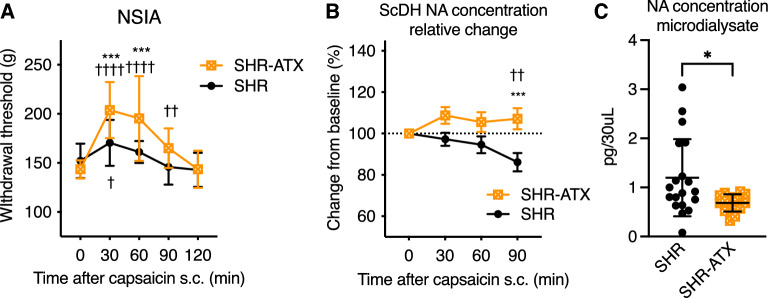
Repeated atomoxetine treatment restored noxious stimuli-induced anesthesia (NSIA) in SHR A standard treatment for ADHD, atomoxetine (0.3 mg/kg/d i.*p*. for 14 d), restored capsaicin-induced NSIA, which is impaired in SHR (**A**). The same atomoxetine treatment restored the capsaicin-evoked spinal noradrenaline increase (**B**) and reduced the spinal basal concentration (**C**). Mean ± SD, *; *p* < 0.05, **; *p* < 0.01, ***; *p* < 0.001 versus SHR, †; *p* < 0.05, ††; *p* < 0.01, ††††; *p* < 0.0001 versus time 0. n = 16 each (**A**), n = 17, 15 (**B**, **C**). ScDH: spinal dorsal horn, NA: noradrenaline, ATX: atomoxetine, POD: postoperative day, SHR: spontaneously hypertensive rats (ADHD model), SHR-ATX: atomoxetine treated SHR.

To examine the effect of standard treatment for ADHD on NSIA, the NA uptake inhibitor atomoxetine (0.3 mg/kg/day) was intermittently administered for 14 days, and capsaicin-induced NSIA was measured (Fig. 4A). This dose of atomoxetine did not clearly alter the ADHD-like behaviors of SHR as described above. This dose of atomoxetine is chosen because it is enough to increase NA in the brain^18^, and to reduce ADHD-like behaviors in different strains^20,21^. Statistical analysis revealed a significant main effect of time after capsaicin (F 4,120 = 31.98, *P* < 0.0001), the effect of atomoxetine treatment (F 2,30 = 9.215, *P* < 0.0001), and interaction (F 4,120 = 7.964, *P* < 0.0001). A post hoc test revealed that atomoxetine-treated SHR had a higher withdrawal threshold at 30 and 60 min after capsaicin injection than non-treated SHR. The change in the spinal NA concentration after capsaicin injection with or without atomoxetine treatment is shown in Fig. 4B. Atomoxetine-treated SHR (SHR-ATX) had a higher spinal NA concentration than non-treated SHR (*P* = 0.0004 at 90 min). Atomoxetine treatment also reduced the basal NA concentration in the spinal microdialysate of SHR compared to non-treated SHR (1.20 ± 0.78 vs. 0.69 ± 0.18 pg /30 μl, *P* = 0.0192, η^2^ = 0.16, Fig. 4C).

### Recovery from incisional pain was delayed in SHR, and roles of alpha2-adrenoceptors

**Figure 5:**
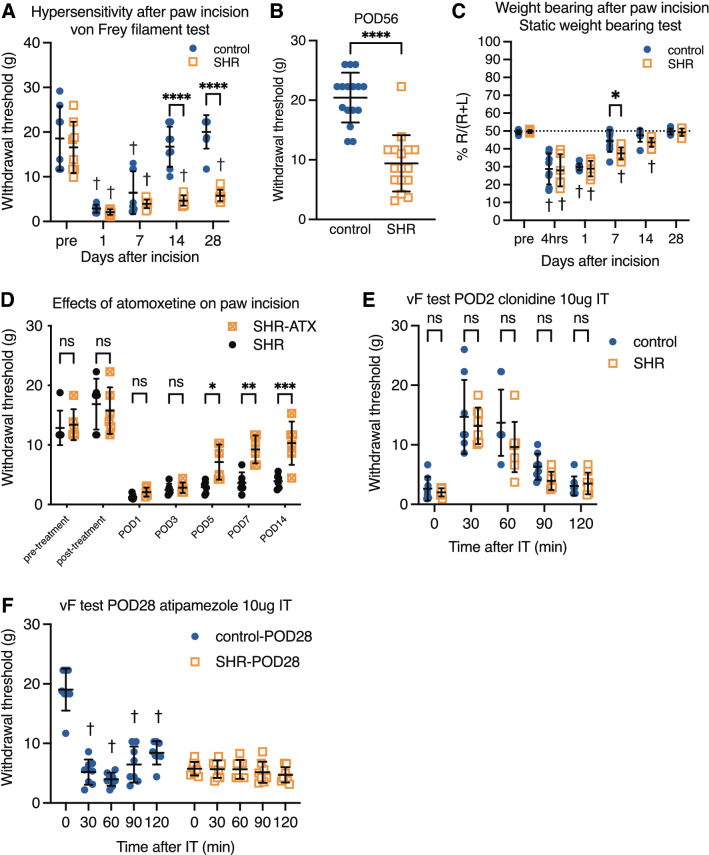
contribution of noradrenergic analgesia to the recovery period after acute incisional pain. The right hind paw incision produced mechanical hypersensitivity in SHR and control. The control animals recovered to pre-surgery values on POD14 (**A**). SHR had a lower withdrawal threshold than the control, even on POD14, POD28, and POD56 (**B**). The static weight-bearing test showed a slight difference between strains on POD7, suggesting that recovery of spontaneous pain behavior after paw incision is slightly delayed in SHR compared with control (**C**). Repeated atomoxetine treatment (0.3 mg/kg/day 14 days) did not change the withdrawal threshold in SHR, but restored delayed recovery after paw incision (**D**). Intrathecal injection of the alpha2-adrenoceptor agonist clonidine (10 μg/10 μl) on the second day after paw incision (POD2) produced anti-hypersensitivity in both SHR and control (**E**). The effect and duration of clonidine did not differ significantly between SHR and control. Intrathecal injection of the alpha2-adrenoceptor antagonist atipamezole (10 μg/10 μl) on POD28 reduced the withdrawal threshold in control, but did not affect SHR (**F**). Mean ± SD, *; *p* < 0.05, ****; *p* < 0.0001 versus control, †; *p* < 0.05 versus pre. †; *p* < 0.05 versus time 0, ns: not statistically significant. n = 8 each (**A**, **C**, **D**, **E**). n = 16 (**B**). vF: von Frey filament test, POD: postoperative day, IT: intrathecal injection. SHR: spontaneously hypertensive rats (ADHD model), SHR-ATX: atomoxetine treated SHR.

To assess the impacts of impaired endogenous analgesia in an acute pain condition, we determined the withdrawal threshold following paw incision. A 2-way ANOVA revealed significant main effects of time after surgery (F 4,72 = 8.23, *P* < 0.0001), strain (F 1,18 = 41.37, *P* < 0.0001), and interaction (F 4,72 = 15.29, *P* < 0.0001). Longitudinal measurements revealed that paw incision produced mechanical hypersensitivity in both SHR and control from POD1 to POD7 (Fig. 5A). The withdrawal threshold of control recovered to the pre-surgery value on POD14, whereas recovery of SHR required more than 14 days), suggesting that pain resolution is delayed in SHR. SHR had a lower withdrawal threshold on POD14 (4.7 ± 1.18 vs. 16.73 ± 4.48 g, *P* < 0.0001) and 28 (5.86 ± 1.24 vs. 20.03 ± 3.77 g, *P* < 0.0001). Measurements from other sets of animals revealed that SHR had a lower withdrawal threshold even on POD56 (9.41 ± 4.71 vs. 20.5 ± 4.18 g, *P* < 0.0001, n = 16 each, η^2^ = 0.62) compared with control, supporting the finding that SHR required more time for recovery (Fig. 5B).

In static weight-bearing experiments, ANOVA revealed significant main effects of time after surgery (F 5,70 = 4.589, *P* < 0.0001), but no effect of strain (F 1,14 = 4.589, *P* = 0.0503) or interaction (F 5,70 = 1.511, *P* = 0.1976). The weight bearing recovered to pre-incision value on POD7 in control and on POD28 in SHR (Fig. 5C). These results suggest that the recovery time of spontaneous pain after paw incision is also delayed in SHR. Recovery from incisional pain was also measured in atomoxetine-treated SHR (SHR-ATX, Fig. 5D). Statistical analysis revealed a significant main effect of time after incision (F 6,90 = 64.20, *P* < 0.0001), effect of treatment (F 2,15 = 26.56, *P* < 0.0001), and interaction (F 12,90 = 4.362, *P* < 0.0001). The post hoc test revealed that SHR-ATX had higher withdrawal threshold than non-treated SHR on POD5, 7, and 14.

To examine the roles of Alpha2AR in the delayed recovery from incisional pain, the Alpha2AR agonist clonidine was injected into the lumbar intrathecal space on POD2 (Fig. 5E) or the Alpha2AR antagonist atipamezole was injected into the lumbar intrathecal space on POD28 (Fig. 5F). Although the use of clonidine or atipamezole in NSIA experiments is important for clarifying the role of Alpha2AR, interpretation is difficult because these drugs directly modify NSIA through Alpha2AR. Therefore, we conducted these experiments after paw incision. A 2-way ANOVA revealed a significant main effect of time after the clonidine injection (F 4,56 = 63.14, *P* < 0.0001), but not of strain (F 1,14 = 1.805, *P* = 0.20) or interaction (F 4,56 = 1.715, *P* = 0.16). Intrathecal clonidine increased the withdrawal threshold in both SHR and control on POD2. This result suggests that Alpha2AR stimulation produces analgesic effects even in SHR. In the intrathecal atipamezole analysis, ANOVA revealed a significant main effect of time after intrathecal atipamezole (F 4,56 = 36.09, *P* < 0.0001), strain (F 1,14 = 51.50, *P* < 0.0001), and interaction (F 4,56 = 33.79, *P* < 0.0001). Intrathecal atipamezole reduced the withdrawal threshold of control, but had no effect on SHR at POD28. These results indicate that Alpha2AR stimulation produces analgesia even in SHR, but the sustained noradrenergic analgesia observed in control during the recovery period is almost completely impaired in SHR. This suggests that SHR may have an abnormality in the noradrenaline releasing process.

## Discussion

The findings of the present study demonstrated impaired descending noradrenergic inhibition and prolonged recovery from acute incisional pain in a rat model of ADHD (SHR). The LC, a major source of NA in the central nervous system, responds to noxious stimulation induced by capsaicin injection, whereas spinal NA does not increase in response to the stimulation. Previous studies in a rat model of chronic neuropathic pain showed that the animals exhibited impaired descending noradrenergic inhibition due to the loss of activation of the LC in response to painful stimulation^22,23^. The present study, however, suggests a new type of impaired descending noradrenergic inhibition due to the dysfunction of the neurotransmitter releasing process. Our present study suggests that pre-existent attenuation of descending noradrenergic inhibition can be the possible reason why ADHD and chronic pain frequently coexist.

SHR have some substrains, and the SHR/NCrl is the most validated animal model of ADHD^24^. Our data showed that the SHR/Izm used in this study also show ADHD-like hyperactivity, inattention, and impulsive behaviors (supplement data), and SHR/Izm partially respond to ADHD treatment with atomoxetine as well as a previous study^7^. We determined that the SHR/Izm reflects the characteristics of ADHD. There is still room for debate about the validity of the model. However, we showed that there might be individuals with impaired endogenous analgesia even if they are not in a chronic pain state. Some previous studies examined pain behaviors in SHR^25–28^. These reports as well as our results suggest that SHR do not show hyperalgesia or allodynia in an untreated state. However, in a condition with painful stimulation that usually activate endogenous analgesia in a separate part of the body, SHR do not show analgesia (Figs. 1A, 2B). This result suggests that noxious stimuli induced analgesia (NSIA) is strongly impaired in SHR. Spinal microdialysis revealed that NA concentration did not increase in the SHR after capsaicin injection (Fig. 1C). NSIA and its similar phenomenon reduce mechanical and thermal hypersensitivity via various neurotransmitters including spinal noradrenaline^29–31^. In SHR, we did not directly validate the roles of spinal noradrenaline in NSIA, however, these results suggest that SHR have dysfunctions in the descending noradrenergic pain inhibitory system.

Immunohistochemistry in the ScDH revealed that the synthesis, the tissue content, and the extracellular concentration of NA, were greater in the SHR (Fig. 2A–D). Excessive NA synthesis may maintain a higher basal extracellular NA concentration to downregulate alpha2-adrenoceptors as shown in Fig. 2H. NA binds alpha2a-adrenoceptors on the spinal neurons and the primary sensory terminals connecting to the spinal neurons. The NA-alpha2a signal decreases excitation or the release of excitatory neurotransmitters to reduce noxious input to the brain. The present results suggest attenuation of NA-Alpha2a-AR mediated analgesia in SHR. NA also elicits analgesia via alpha1-adrenoceptors^32,33^. In addition, alpha1-adrenoceptors activated by the sympathetic nervous system are pronociceptive^34^, and SHR express a higher level of alpha1-adrenoceptors^35^. The role of alpha1-adrenoceptors in pain processing in SHR requires further study. Conversely, SHR showed greater amounts of NET in the ScDH, which can rapidly reduce the NA concentration after its release (Fig. 2E,F). However, increased spinal NETs did not contribute to extracellular NA concentration in conditions without pain (Fig. 2). However, the impact of NA uptake via the increased NET can be significant under conditions in which excess NA is released in response to painful stimuli that normally elicit NSIA. The spinal mechanisms by which NSIA is attenuated are thought to be the downregulation of Alpha2aAR and an imbalance in NA imbalance of NA synthesis/reuptake.

As shown above, the increase of spinal NA concentration during NSIA in SHR completely vanished. However, immunohistochemistry revealed that pERK and c-fos expression as markers of neuronal activation increased after capsaicin injection in the LC of both SHR and control (Fig. 3). These results suggest that LC activity in response to noxious stimulation is reserved in SHR, but NA is not released in the ScDH. Strong autoinhibition via alpha 2a-adrenoceptors on the axon terminals of noradrenergic neurons in the ScDH may suppress NA release from the terminals. However, spinal alpha 2a-adrenoceptors were reduced in SHR than control. Excessive uptake via NET may also reduce the spinal NA concentration. These two mechanisms probably do not explain the almost-zero increase in spinal NA after strong LC activation. Our experiment using intrathecal clonidine injection revealed that alpha2-adorenoceptor activation produce analgesia in SHR (Fig. 5E). This result suggests that some abnormalities in the spinal neurotransmitter releasing process may be present in SHR. Some previous studies reported that patients with ADHD have polymorphisms in the genes related to neurotransmitter release, like SNARE (soluble *N*-ethylmaleimide-sensitive factor attachment protein receptor)^36^ or synaptotagmin^37^. Whether these proteins regulate NA release needs to be investigated in the future, but they may be involved in the abnormal NA release in the spinal cord observed in the present study.

Impaired NSIA in SHR can be explained by loss of spinal NA increase. However, immunohistochemistry experiments in LC suggest NA can be released in other brain regions, and the brain NA can also be involved in altered pain modulation. To explore this question, we examined noradrenergic systems including alteration of NA concentration in the mPFC after painful stimulation (supplement Fig. 3). These results suggest that SHR have alterations in NA concentration and releasing process similar to the ScDH. There abnormalities can be related to the ADHD symptoms, they can also facilitate pain directly^38^ or indirectly by aversion^39^ or depression^40^ or diversion^28^. A previous study reported that chronic pain animals also showed elevated concentration and loss of responsive increase of NA in the mPFC^16^. In this way, our study at least revealed that chronic pain and ADHD might share features of abnormal noradrenergic modulation of the brain.

Furthermore, in our present study, standard ADHD treatment, atomoxetine, partially restored the Y-maze score (supplement Fig. 2C). The SHR-ATX group showed restored NSIA and enhanced recovery after paw incision (Figs. 4A, 5D). Atomoxetine increases noradrenaline concentration in the central nervous system immediately after the administration in SHR^41,42^. However, in this study, atomoxetine treatment reduced the basal noradrenaline concentration and restored the capsaicin-evoked spinal release of noradrenaline (Fig. 4B, 4C). We measured spinal noradrenaline concentrations the day after the last atomoxetine administration. Therefore, no increase in noradrenaline concentration was observed. This result also suggests that atomoxetine may improve NSIA by restoring the neurotransmitter release-related dysfunction rather than effects on noradrenaline concentration by reuptake inhibition. Reduction of basal NA may attenuate the downregulation of alpha 2a-adrenoceptors in the ScDH as a target of descending inhibitory systems. Atomoxetine (0.3–3.0 mg/kg) is reported to attenuate ADHD-like behaviors in SHR^6,43,44^. A previous preclinical study reported that atomoxetine reduces NET levels in the brain^45^. Furthermore, the study reported that atomoxetine alters SNARE proteins and N-methyl-D-aspartate receptor levels in the brain^45^. Atomoxetine also upregulates the expression of BDNF and signaling in the brain^46^. Our previous studies revealed that BDNF-TrkB signaling normalizes LC dysfunction and induces spinal noradrenergic and cholinergic plasticity to enhance analgesia^29,47^. Atomoxetine may have similar effects on SHR.

The impaired NSIA in SHR are associated with the delayed recovery of acute pain. After the paw incision surgery, SHR exhibited the same level of acute hypersensitivity and weight bearing as control, but required more time to recover (> 28 days) (Fig. 5A–C). Spinal noradrenergic signaling is necessary to recover from acute incisional pain^29,47–49^. Our experiment using intrathecally injected atipamezole (Fig. 5F) also suggested that spinal noradrenergic analgesia does not work in SHR during recovery after surgery (POD28). Some previous studies reported that chronic neuropathic pain impairs descending noradrenergic inhibition and produces pain chronification of additional acute pain^29,48^. Our present study suggests the existence of a population with impaired endogenous analgesia even without persistent pain, and that the group has a risk factor for prolonged pain after surgery. In pediatric patients, acute pain scores after surgery are not significantly different between patients with or without ADHD^50^. There are no systemic review articles regarding acute and chronic pain scores after surgery in adult ADHD patients. Impaired pain modulation is a risk factor for chronic postsurgical pain^51–53^. An ADHD diagnosis is not listed as a risk factor for chronic postsurgical pain, but considering the high frequency of the co-existence of ADHD and chronic pain, it is possible that undiagnosed adult ADHD patients may experience prolonged pain after surgery. Furthermore, impaired pain modulation is observed in various types of chronic pain^54^. Especially, painful conditions categorized as nociplastic pain, such as fibromyalgia, are associated with decreased endogenous analgesia^55,56^. Fibromyalgia patients are more likely to have ADHD^3^. Accordingly, SHR may reflect at least some features of nociplastic pain.

In conclusion, ADHD model rats showed impaired descending noradrenergic inhibition and acute pain chronification, which may be due to a synthesis-reuptake imbalance and dysfunction of NA releasing processes in the ScDH. Repeated atomoxetine treatment restored the descending inhibition and pain chronification. Impaired noradrenergic pain modulation may be a common neurologic alteration between ADHD and chronic pain patients.

## Methods

### Ethical perspective

The study was performed in accordance with Guide for the Care and Use of Laboratory Animals. All procedures were approved by the Animal Care and Use Committee of Gunma University (Maebashi, Japan. No. 17-071, 21-069). The study was carried out in compliance with the ARRIVE guidelines.

### Animals

Spontaneously hypertensive rats (SHR/Izm, n = 144) and their normotensive controls (Wister Kyoto: WKY/Izm, n = 123) were supplied by the Disease Model Cooperative Research Association (Kyoto, Japan). Adult male and female rats, 6 to 7 weeks old (210–240 g) at the time of paw incision surgery, were housed under a 12-h light–dark cycle, and fed ad libitum. The animals were randomly assigned to each experimental group (SHR, SHR-ATX, and control). In the SHR-ATX group, 5 weeks old SHR were injected with atomoxetine (0.3 mg/kg) intraperitoneally once a day for 14 days. Atomoxetine hydrochloride (ATX) was purchased from Tokyo Chemical Industry Co., Ltd. (Tokyo, Japan) and was dissolved in saline (0.3 mg/ml). The following experiments were performed at an approximately 24-h interval from the last atomoxetine injection.

### Validation of ADHD-like behaviors in SHR

Open field test and Y-maze test were conducted to validate the behaviors of SHR as a model of ADHD. Open-field test was performed to evaluate the locomotor activity and anxiety. Rats were placed in a cage (60 cm × 60 cm × 60 cm) in an open field apparatus. The light intensity was equal (150 lx) in all parts of the field. Animals were acclimated to the experimental room for 30 min prior to the start of the behavioral session. Rats were placed in the center of the open field and allowed to explore freely for 10 min. The maze was thoroughly cleaned after each rat to attenuate olfactory trails. The locomotion during the test was recorded and calculated using a software (TimeFZ, O’Hara & Co.,ltd., Tokyo, Japan). The total distance was presented as pixels. The Center area was set as 50% of the total area of the floor of the open field. Time spent in the center area was presented as frames.

Y-maze test was performed to evaluate the locomotor activity and inattention or short term working memory of SHR. This test was conducted in a plastic mat black-colored Y-maze. The Y-maze consisted of three arms made of black plastic (50 cm long, 20 cm high, 10 cm wide) extending from a central platform at an angle of 120°. The light intensity was equal (150 lx) in all parts of the field. A camera recorded the sessions, which were scored at a later time. Animals were acclimated to the experimental room for 30 min prior to the start of the behavioral session. Rats were placed in the center of the Y-maze and allowed to explore freely for 8 min. The maze was thoroughly cleaned after each rat to attenuate olfactory trails. The sequence of arm entries was recorded manually from the recordings. An arm entry was defined as the entry of half of the body trunk into one arm. Alternation was defined as multiple entries into the three different arms in overlapping triplet sets. The percentage of spontaneous alternation was calculated as the ratio of the actual-to-possible alternations (defined as the total number of arm entries − 2) multiplied by 100. Spontaneous alternations are the number of three consecutive entries into three different arms (A, B, C), such as ABC, ACB, BAC, BCA, CAB, or CBA during the test session. The same arm return is defined as an arm entry to the same arm, such as AA, BB, and CC. The performance of an animal was excluded if the total number of arm entries was less than 7.

### Noxious stimuli-induced analgesia (NSIA)

We assessed NSIA in naïve rats as previously described^57^. Briefly, the withdrawal threshold in the left hind paw was measured using the paw pressure test. Under brief isoflurane anesthesia (2% in oxygen), capsaicin (150 µg/50 µl) was injected into the left forepaw, and the withdrawal threshold in the left hind paw was measured at 30-min intervals after the injection.

### Microdialysis in the spinal cord dorsal horn (ScDH)

Microdialysis in the ScDH was performed as described previously^29^. Briefly, SHR or control were anesthetized with 2.0% isoflurane, and anesthesia was maintained with 1.5% isoflurane in 100% oxygen during the measurements. The rectal temperature of the animals was maintained at 37 °C using a heating pad. Saline was infused at a rate of 1 mL/h through a cannulated left tail vein using an infusion pump system (Fusion 400, Chemyx, Stafford, TX, USA). The L4-L6 spinal cord was exposed by a T13-L1 laminectomy. Microdialysis probes (CX-I-8-01, Eicom Co., Kyoto, Japan) were inserted into the right ScDH and perfused with Ringer’s solution (147 mmol/L NaCl, 4 mmol/L KCl, 2.3 mmol/L CaCl_2_) at a rate of 1 µl/min. After 60 min of constant perfusion, dialysates were collected at 30-min intervals. After collecting two dialysates for baseline samples, capsaicin (150 μg/50 μl) was injected subcutaneously into the left forepaw. The noradrenaline (NA) concentration was measured for 90 min after the capsaicin injection using an HTEC-500 high-performance liquid chromatography (HPLC)-electrochemical detection system (Eicom Co.). The basal NA concentrations are presented as the mean of two 30-min dialysate measurements. The change in the NA concentration after capsaicin injection is presented as a percentage compared with the baseline measurement (100%).

### Tissue content of NA

For NA measurements, the animals were decapitated under deep anesthesia, and the spinal cord was quickly harvested. The left portion of the lumbar enlargement was immediately dissected into 4-mm lengths of the dorsal horn and weighed. The samples were homogenized in 1000 µl of homogenization solution (0.2 M perchloric acid containing 0.1 mM EDTA-2Na, 10 ng/ml isoproterenol as an internal standard) and centrifuged at 20,000 g at 0 °C for 15 min. The supernatants were filtered through a centrifugal filter with a 0.45-µm pore (PALL, Puerto Rico) at 2400 g at 0 °C for 15 min, and adjusted to pH 3.5 with 1 M sodium acetate. Samples (10 µl) were injected into an HPLC-electrochemical detection system (HTEC-500). The chromatographic conditions were as follows: The mobile phase comprised 0.1 M phosphate buffer (pH 6.0) containing 5 mg/l EDTA-2Na, 175 mg/l sodium 1-octane sulfonate acid, and 17% methanol. The column was an EICOMPAK SC-5ODS column (3.0 × 15 mm, Eicom Co).

### Immunohistochemistry

Rats were killed by an intraperitoneal injection of pentobarbital (50 mg/kg body weight) and perfused with 0.01 M phosphate-buffered saline (PBS) containing 1% sodium nitrite, followed by 4% paraformaldehyde in 0.1 M PBS. The brains and spinal cords were dissected out, post-fixed, and cryoprotected. The spinal cord including lumber enlargement was sectioned at a 20-µm thickness. The sections were incubated with a mouse monoclonal anti-dopamine-β-hydroxylase (DbH) antibody (1:500, Millipore, MAB308, RRID: AB_2245740), a mouse monoclonal anti-NA transporter (NET) antibody (1:1000, MAb Technologies Cat# NET05-2, RRID: AB_2571639), a rabbit anti–alpha2-adrenoceptor antibody (1:1000, Neuromics Cat# RA14110-150, RRID: AB_2225052), or a rabbit anti-iba1 antibody (1:1000, FUJIFILM Wako Chemicals Cat# 019–19,741, RRID: AB_839504) for 48 h at 4 °C, followed by the corresponding secondary antibody, Cy2 conjugated anti-mouse IgG (1:200, Jackson Immuno Research Laboratories, West Grove, PA, 711-155-152), Cy3 conjugated anti-rabbit IgG (1:600, Jackson Immuno Research Laboratories, 705-225-147), or Alexa-Fluor647 conjugated anti-rabbit IgG (1:500, Jackson Immuno Research Laboratories, 711-605-152). Finally, the sections were dehydrated in ethanol, cleaned in xylene, and cover-slipped with DPX mounting medium (Millipore Sigma) at room temperature.

Twelve-bit images were captured using a fluorescence microscope (eclipse Ni-E, Nikon Co., Tokyo, Japan) fitted with a digital camera (Andor Zyla 5.5, Oxford Instruments, UK) using a 40 × or 100 × objective at a resolution of 2160 × 2560 pixels. Each type of immunostaining was quantified in 3–4 randomly selected sections from each animal with a 250 × 250 pixel region of interest set in the middle of the ScDH. The area fraction of immunoreactive pixels determined by constant optical threshold was measured using Fiji-ImageJ software^58^. The individual performing the image quantification was blinded to the treatment.

### Immunohistochemistry of pERK and c-fos in the LC

As previously described, capsaicin-evoked neuronal activation of the noradrenergic LC neurons was examined with immunohistochemistry^23,47^. Under brief isoflurane anesthesia (2%), capsaicin, at the same dose used for NSIA (150 µg/50 µl; 50% ethanol in saline), or vehicle was injected into the left forepaw. At 30 or 60 min later, rats were killed by an intraperitoneal injection of pentobarbital (50 mg/kg) and perfused with 0.01 M PBS containing 1% sodium nitrite, followed by 4% paraformaldehyde in 0.1 M PBS. The brainstems were dissected out, post-fixed, cryoprotected, and sectioned at a 20-µm thickness. The sections were incubated with a rabbit monoclonal anti–c-fos antibody (1:1000, Santa Cruz Biotechnology Cat# sc-253, RRID: AB_2231996) or a rabbit monoclonal anti-pERK antibody (1:600, Cell Signaling Technology Cat# 4370, RRID: AB_2315112) and a mouse monoclonal anti-DbH antibody (1:1000, Millipore, MAB308, RRID: AB_2245740), followed by Alexa-Fluor647 conjugated anti-rabbit IgG (1:500, Jackson Immuno Research Laboratories, 711-605-152), Cy2 conjugated anti-mouse IgG (1:200, Jackson Immuno Research Laboratories, 711-155-152), and incubated with DAPI solution (4′,6-diamidino-2-phenylindole dihydrochloride, 1:20,000, Thermo Fisher Scientific, Pittsburgh, PA, D1306, RRID: AB_2629482). Finally, the sections were dehydrated in ethanol, cleaned in xylene, and cover-slipped with DPX mounting medium (Sigma-Aldrich) at room temperature.

Twelve-bit images were captured using a fluorescence microscope with a 20 × objective at a resolution of 2160 × 2560 pixels. Three or four randomly selected sections from each animal were used. The DbH-immunoreactive (IR) area was defined as the LC area. Artificial intelligence-based segmentation Fiji-Unet plug-in for Fiji-ImageJ^59^ was used to produce the DAPI mask. Using the DAPI mask, the mean gray value of the pERK or c-fos channel was measured using Fiji-ImageJ software^58^. The individual performing the image quantification was blinded to the treatment.

### Paw incision

A paw incision was performed on the right hind paw as previously described^60^. Briefly, animals were anesthetized with 2.0% isoflurane in oxygen. A 1-cm midline incision was made using a No. 11 surgical blade at 0.5 cm from the proximal edge of the heel. The plantaris muscle was elevated and incised longitudinally. After the incision, the wound was closed with 5-0 nylon mattress sutures. Mechanical hypersensitivity was measured before and at 1, 3, 5, 7, 14, 28, and 56 days after the incision as described below. The sutures were removed after the measurement on the third day. In the SHR-ATX group, the incision was made on the next day of the last ATX administration and they did not receive ATX administration on the following days.

### Testing the mechanical hypersensitivity after paw incision

Mechanical hypersensitivity was determined using 8 von Frey filaments (Stoelting, Wood Dale, IL, USA) ranging from 0.6 to 26 g, as previously described^61^. Briefly, rats were placed in individual acrylic chambers with a plastic mesh floor and allowed to acclimate to the environment for at least 30 min prior to the test. The filaments were then applied to the heel of the hind paw to the bending point for 6 s. A brisk paw withdrawal was considered to be a positive response. The up-down method was used to determine the withdrawal threshold. The investigator performing the behavioral tests was blinded to the group.

### Static weight-bearing test

Assessment of pain-related behaviors was also performed using a hindlimb weight-bearing apparatus (BIO-SWB-TOUCH, Bioseb, Vitrolles, France) as reported previously with minor modification^62^. Rats were positioned in the chamber of the apparatus and allowed to adapt for at least 5 min. The training session was conducted 2–3 times prior to the paw incision surgery, postoperative day (POD) 14, and POD28. When stationary, readings were taken over 5 s. The percent weight borne on the right leg was determined according to the following formula:

$$\begin{gathered} \% {\text{ Weight }}\;{\text{of}}\;{\text{ right }}\;{\text{leg }}\left( {\% {\text{R}}/\left( {{\text{R}} + {\text{L}}} \right)} \right) \hfill \\ = \, \left[ {{\text{weight }}\;{\text{of }}\;{\text{right}}\;{\text{ leg}}/ \, \left( {{\text{weight}}\;{\text{ of }}\;{\text{right}}\;{\text{ leg }} + {\text{ weight of left}}\;{\text{ leg}}} \right)} \right]*{1}00 \hfill \\ \end{gathered}$$

A total of 5 readings were obtained for each rat at each time-point, and the average of the 5 readings was calculated and used for subsequent analyses.

### Intrathecal injections

To evaluate the roles of spinal noradrenergic signaling on acute pain threshold, we performed intrathecal injections of clonidine hydrochloride (α2 adrenoceptor agonist, Fujifilm Wako Pure Chemicals, Tokyo, Japan) or atipamezole hydrochloride (α2 adrenoceptor antagonist, Fujifilm Wako Pure Chemicals, Tokyo, Japan) on POD2, 28 respectively. Drugs were dissolved in saline. Under anesthesia with 2% isoflurane, clonidine (10 µg/10 µl) or atipamezole (10 µg/10 µl) was injected intrathecally through the L5/6 intervertebral space using a 30-gauge needle.

### Statistical analysis

We performed a power analysis for the primary outcome (NSIA) to determine the appropriate sample size. The analysis assumed a mean difference of 50 g in the withdrawal threshold in the left hind paw and a standard deviation of 30 g in each group according to the results of a previous study^48^. We determined that the use of more than 7 rats in each group would result in the detection of significant differences with 80% power at a significant level of α = 0.05. The sample size of spinal microdialysis was determined based on the power analysis assuming a mean difference of 20% in the change of concentration and a standard deviation of 20 in each group. We determined that the use of more than 17 rats in each group would result in the detection of significant differences with 80% power at a significant level of α = 0.05.

The behavioral testing data are presented as mean ± standard deviation (SD). The behavioral testing, microdialysis, and immunostaining data were analyzed by 2-way analysis of variance (ANOVA) followed by Student’s t-test with Bonferroni’s or Dunnett’s correction method using Prism 9 software (GraphPad Software, San Diego, CA, version 9.5.0) or R software (R Foundation for Statistical Computing). The strain (SHR and control) and time (time after capsaicin injection or surgery) were considered independent variables. ANOVA results are presented as the value of the Fisher distribution (Fx,y) obtained from the data, where x is the degrees of freedom for the variable and y is the total degrees of freedom of the distribution. Other results were analyzed using the Student’s t-test. The levels of significance were set as *p* < 0.05.

## Supplementary Information

Supplementary Information.

## Data Availability

The datasets used and/or analyzed during the current study are available from the corresponding author upon reasonable request.
