# Supplementary material for: Rat model of attention-deficit hyperactivity disorder exhibits delayed recovery from acute incisional pain due to impaired descending noradrenergic inhibition
Source: Sci Rep. 2023 Apr 4;13:5526. doi: 10.1038/s41598-023-32512-9 (PMC10073110; doi:10.1038/s41598-023-32512-9)
Supplement: Supplementary file 1 — Supplementary Information. [file 41598_2023_32512_MOESM1_ESM.docx]

S1 Validation of ADHD-like behaviors of SHR

S1.1.1 Open filed test

Rats were placed in a cage (60cm×60cm×60cm) in an open field apparatus. The light intensity was equal (150 lx) in all parts of the field. Animals were acclimated to the experimental room for 30 min prior to the start of the behavioral session. Rats were placed in the center of the open field and allowed to freely explore for 10 min. The maze was thoroughly cleaned after each rat to attenuate olfactory trails. The locomotion during the test was recorded and calculated using a software (TimeFZ, O’hara & Co.,ltd., Tokyo, Japan). Total distance was presented as pixels. Center area was set as 50% of the total area of the floor of the open-field. Time spent in the center area was presented as frames.

S1.1.2 Results

To validate SHR/Izm as an animal model of ADHD, open-field test was performed to evaluate the locomotor activity and anxiety. SHR and SHR-ATX showed longer locomotion during the test than control (1450 ± 584.7 vs. 1491 ± 221.5 vs. 1016 ± 311.0 px., Supplement Figure 1A). One-way ANOVA revealed the significant effect (F 2,39 = 5.97, *P* = 0.0055) and post-hoc test show significant difference between SHR and control (P = 0.02). Repeated atomoxetine treatment did not alter the distance (SHR vs. SHR-ATX, P > 0.999). SHR and SHR-ATX showed longer duration spent in the center area than control (170 ± 198 vs. 743.4 ± 390 vs. 733.2 ± 357 frames, Supplement Figure 1B). One-way ANOVA revealed the significant effect (F 2,39 = 14.15, *P* < 0.0001) and post-hoc test show significant difference between SHR and control (P < 0.0001). Repeated atomoxetine treatment did not alter the duration in the center area (SHR vs. SHR-ATX, P > 0.999).

Supplement figure 1. Open-field test

SHR showed longer locomotion during the open-field test than control(A). SHR also showed longer duration spent in the center area than control (B). these results suggest that SHR/Izm reflect hyperactive and impulsive aspects of ADHD. Repeated atomoxetine (0.3mg/kg 14days) treatment to SHR did not change the total distance and time spent in the center area.

Mean ± SD, ****; p < 0.0001, *; p < 0.05, ns; not significant, n = 14 each

control: Wister Kyoto, SHR: spontaneously hypertensive rats (ADHD model), SHR-ATX: atomoxetine treated SHR

S1.2.1 Y-maze test

This test was conducted in a plastic mat black-colored Y-maze. The Y-maze consisted of three arms made of black plastic (50 cm long, 20 cm high, 10 cm wide) extending from a central platform at an angle of 120°. The light intensity was equal (150 lx) in all parts of the field. A camera recorded the sessions, which were scored at a later time. Animals were acclimated to the experimental room for 30 min prior to the start of the behavioral session. Rats were placed in the center of the Y-maze and allowed to freely explore for 8 min. The maze was thoroughly cleaned after each rat to attenuate olfactory trails. The sequence of arm entries was recorded manually from the recordings. Arm entry was defined as the entry of half of the body trunk into one arm. Alternation was defined as multiple entries into the three different arms in overlapping triplet sets. The percentage of spontaneous alternation was calculated as the ratio of the actual-to-possible alternations (defined as the total number of arm entries − 2) multiplied by 100. Spontaneous alternations are the number of three consecutive entries into three different arms (A, B, C) such as ABC, ACB, BAC, BCA, CAB, or CBA during the test session. The same arm return is defined as a arm entry to the same arm such as AA, BB, CC. The performance of an animal was excluded if the total number of arm entries was less than 7.

S1.2.2 Results

To validate SHR/Izm as an animal model of ADHD, Y-maze test was performed to evaluate the locomotor activity and inattention or short term working memory. There were no difference between SHR or SHR-ATX and control in spontaneous alternation or alternate arm return (61.34 ± 7.1 vs. 69.78 ± 10.5 vs. 62.91 ± 12.4 %., Supplement Figure 2A, 28.13 ± 7.5 vs. 26.4 ± 10.2 vs. 30.56 ± 8.5 %., Supplement Figure 2B). One-way ANOVA revealed no significant effect (F 2,41 = 2.91, *P* = 0.065, F 2,41 = 0.82, *P* = 0.44). Repeated atomoxetine treatment did not alter these alternation behaviors. However, SHR showed more same arm return than control and SHR-ATX (1[0-1] vs. 0 [1-2]vs. 0 [0-1]times, Supplement Figure 2C). Kruskal-Wallis test revealed significant effect (*P* = 0.0011), and multiple comparison revealed SHR showed more same arm return than control (P = 0.0039) and atomoxetine treatment reduce the time (SHR vs. SHR-ATX, P = 0.0038).

SHR and SHR-ATX showed more time of arm entry than control (SHR: 20[19-24] vs. SHR-ATX: 21.5[19-23] vs. control 16[13-20]). Kruskal-Wallis test revealed significant effect (*P* = 0.0024), and multiple comparison revealed SHR showed more time of arm than control (P = 0.0059) and atomoxetine treatment did not reduce the time (SHR vs. SHR-ATX, P > 0.99).

Supplement figure 2. Y-maze test

The ratio of spontaneous alteration and alternate arm return are not different among SHR, SHR-ATX, and control (A,B). SHR showed more attempts of the same arm return (C) and total arm entry (D). These results suggest that SHR/Izm reflect hyperactivity and inattention aspects of ADHD. Repeated atomoxetine (0.3mg/kg 14days) treatment to SHR did not change the spontaneous alteration and alternate arm return, but decreased the time of the same arm return and total arm entry.

Mean ± SD (A,B), Median ± 95%CI (C,D) ***; p < 0.001, **; p < 0.01, ns; not significant, n = 14,14,16

control: Wister Kyoto, SHR: spontaneously hypertensive rats (ADHD model), SHR-ATX: atomoxetine treated SHR

S2 NA release from noradrenergic neurons projecting to the mPFC

S2.1.Microdialysis in the medial prefrontal cortex (mPFC)

Cannulation of the mPFC was performed 5–7 days prior to the microdialysis as previously reported.^16^ Animals were anesthetized with 2.0% isoflurane in 100% oxygen, and anesthesia was maintained with 1.0-1.5% isoflurane. The animal was placed securely in a stereotaxic frame (KOPF, Tujunga, CA), and a sterile stainless-steel guide cannula (CGX-6, Eicom Co., Kyoto, Japan) was implanted into the right mPFC (3.0 mm anterior and 0.5 mm lateral to the bregma, and 3–4.0 mm ventral from the surface of the dura mater) according to a rat brain atlas.^56^ Under isoflurane anesthesia, a microdialysis probe (outer diameter = 0.22 mm, inner diameter = 0.20 mm, membrane length = 2 mm; EICOM Co. CX-I-6-02) was inserted through the guide cannula and perfused with Ringer’s solution at a constant flow rate (1 µl/min) using a syringe pump (ESP-64; EICOM Co.). Dialysates were collected every 30 min, and the NA concentration was analyzed in the same manner as for the ScDH microdialysis.

S2.2 mPFC Immunohistochemistry

Rats were killed by an intraperitoneal injection of pentobarbital (50 mg/kg body weight) and perfused with 0.01 M phosphate-buffered saline (PBS) containing 1% sodium nitrite, followed by 4% paraformaldehyde in 0.1 M PBS. The brains and spinal cords were dissected out, post-fixed, cryoprotected. The PFC tissue was sectioned at a 20-µm thickness. The sections were incubated with a mouse monoclonal anti-dopamine-β-hydroxylase (DbH) antibody (1:500, Millipore, MAB308, RRID: AB_2245740), a mouse monoclonal anti-NA transporter (NET) antibody (1:1000, MAb Technologies Cat# NET05-2, RRID: AB_2571639), a rabbit anti–alpha2-adrenoceptor antibody (1:1000, Neuromics Cat# RA14110-150, RRID: AB_2225052), or a rabbit anti-iba1 antibody (1:1000, FUJIFILM Wako Chemicals Cat# 019-19741, RRID: AB_839504) for 48 h at 4°C, followed by the corresponding secondary antibody, Cy2 conjugated anti-mouse IgG (1:200, Jackson Immuno Research Laboratories, West Grove, PA, 711-155-152), Cy3 conjugated anti-rabbit IgG (1:600, Jackson Immuno Research Laboratories, 705-225-147), or Alexa-Fluor647 conjugated anti-rabbit IgG (1:500, Jackson Immuno Research Laboratories, 711-605-152). Finally, the sections were dehydrated in ethanol, cleaned in xylene, and cover-slipped with DPX mounting medium (Millipore Sigma) at room temperature.

Twelve-bit images were captured using a fluorescence microscope (eclipse Ni-E, Nikon Co., Tokyo, Japan) fitted with a digital camera (Andor Zyla 5.5, Oxford Instruments, UK) using a 40x or 100x objective at a resolution of 2160 x 2560 pixels. Each type of immunostaining was quantified in 3–4 randomly selected sections from each animal with a 250x250 pixel region of interest set in the middle of the medial PFC. The area fraction of immunoreactive pixels determined by constant optical threshold was measured using Fiji-ImageJ software. The individual performing the image quantification was blinded to the treatment.

S2.3 Results

The spinal NA did not increase after painful stimulation, but the LC of SHR was activated. To investigate whether this mismatch is global or spinal cord specific, the noradrenergic system of mPFC was examined.

Like in the spinal cord, the NA concentration in the mPFC was higher in SHR than in control (1117 ± 196.4 vs. 920.8 ± 149.3 pg/mg tissue, *P*= 0.041, η^2^ = 0.27) (Figure 5A). In contrast, the DbH-IR area in the mPFC did not differ between the strains (2.06 ± 0.58 vs. 2.32 ± 0.37 %, *P*= 0.312, η^2^ = 0.07) (Figure 5B). The NET-IR area and the Alpha2aAR-IR area were higher in SHR than in control (2.98 ± 1.48 vs. 1.40 ± 0.80 %, *P*= 0.0196, η^2^ = 0.033, Figure 5C, 15.8 ± 5.7 vs. 8.74 ± 5.4 %, *P*= 0.024, η^2^ = 0.31, Figure 5D).

To assess the functional differences in noradrenergic neurons between the strains, changes in the NA concentration in the mPFC were determined after painful stimulation induced by capsaicin injection (Figure 5E). Statistical analysis revealed a significant main effect of time after capsaicin (F 3,111 = 4.412, *P*= 0.0057), but no significant effect of strain (F 3,37 = 2.495, *P*= 0.075), or interaction (F 9,111 = 1.463, *P*= 0.1707). The post hoc test revealed that the NA concentration in the mPFC of control was higher at 60 and 90 min after capsaicin injection than at baseline, but no statistical difference was seen in SHR. These results indicate that the mismatch of LC activation and loss of NA elevation at the axon terminal is observed in mPFC as well as the ScDH. Excessive reuptake via NET or autoinhibition of NA release by Alpha2aAR is the possible mechanism of this difference.

Supplement figure 3. The difference in noradrenergic systems of the medial prefrontal cortex (mPFC) between SHR and control.

The dopamine-β-hydroxylase (DbH) immunoreactive (IR) area in the mPFC was not statistically different between the strains (A). The noradrenaline transporter (NET) IR area (B) and the alpha2a-adrenoceptor (Alpha2a-AR) IR area in the mPFC were higher in SHR (C). The noradrenaline concentration of homogenate from the mPFC was higher in SHR (D). Repeated atomoxetine treatment (0.3mg/kg/day) did not increase NA concentration in mPFC. The noradrenaline concentration of microdialysate from the mPFC was higher in SHR (E). Change in the noradrenaline concentration in microdialysates from the mPFC after capsaicin stimulation are shown in (F). Painful capsaicin injection increased NA in mPFC of control, but not in SHR.

Mean ± SD, *; p < 0.05 vs. control, ###; p< 0.001 vs time 0, ns: not significant, n = 8 each.

control-cap: capsaicin injected WKY, control-veh: vehicle injected WKY, SHR-cap: capsaicin injected SHR, SHR-veh: vehicle injected SHR.
